# Accurate Ambient Noise Assessment Using Smartphones

**DOI:** 10.3390/s17040917

**Published:** 2017-04-21

**Authors:** Willian Zamora, Carlos T. Calafate, Juan-Carlos Cano, Pietro Manzoni

**Affiliations:** 1 Department of Computing Engineering, Universitat Politècnica de València, 46022 València, Spain; calafate@disca.upv.es (C.T.C.); jucano@disca.upv.es (J.-C.C.); pmanzoni@disca.upv.es (P.M.); 2 School of Computer Science, Universidad Laica Eloy Alfaro de Manabí, 130802 Manta, Ecuador; willian.zamora@live.uleam.edu.ec

**Keywords:** crowdsensing, smartphone, noise sensing, dynamic range

## Abstract

Nowadays, smartphones have become ubiquitous and one of the main communication resources for human beings. Their widespread adoption was due to the huge technological progress and to the development of multiple useful applications. Their characteristics have also experienced a substantial improvement as they now integrate multiple sensors able to convert the smartphone into a flexible and multi-purpose sensing unit. The combined use of multiple smartphones endowed with several types of sensors gives the possibility to monitor a certain area with fine spatial and temporal granularity, a procedure typically known as crowdsensing. In this paper, we propose using smartphones as environmental noise-sensing units. For this purpose, we focus our study on the sound capture and processing procedure, analyzing the impact of different noise calculation algorithms, as well as in determining their accuracy when compared to a professional noise measurement unit. We analyze different candidate algorithms using different types of smartphones, and we study the most adequate time period and sampling strategy to optimize the data-gathering process. In addition, we perform an experimental study comparing our approach with the results obtained using a professional device. Experimental results show that, if the smartphone application is well tuned, it is possible to measure noise levels with a accuracy degree comparable to professional devices for the entire dynamic range typically supported by microphones embedded in smartphones, i.e., 35–95 dB.

## 1. Introduction

Noise is considered a particular type of environmental pollutant since, at certain levels, it can affect people both physiologically and psychologically and, more important, also interfere with basic activities, such as sleep, rest, study, communication and socializing [1,2]. In fact, different studies [3,4] have highlighted the importance of noise control in highly-populated areas. Similarly, the European Environment Agency has been enacting new regulations for the evaluation and control of environmental noise [5,6], and all major cities have their own regulation.

The traditional approach to measuring acoustic pollution is through the use of professional sound level meters, which are of considerable cost and size, having high accuracy and sensitivity. Usually, these measurements are taken at a limited number of places and are then processed using different statistical techniques to generate acoustic pollution maps for well-defined target areas, thereby providing finer spatial granularity.

Recently, crowdsensing solutions have emerged as a promising solution by promoting collaborative monitoring of populated areas. The basic concept behind crowdsensing is that all users contribute to the same goal by measuring some environmental variable, and then, the different measurements are stored on a server and processed using big data techniques for data merging and analysis. Furthermore, the rapid adoption of mobile phones, together with the increasing number of sensors these devices are equipped with (e.g., high-quality cameras, microphone and accelerometers), greatly simplifies the widespread adoption of crowdsensing solutions, reducing the hardware requirements and costs to a minimum. These factors have led to a remarkable growth of crowdsensing proposals both from academia and from industry, e.g., [4,7,8,9]. In particular, participatory solutions, such as NoiseTube [10], Ear-Phone [11] and NoiseSPY [4], use smartphones to measure environmental noise levels in urban areas, generating pollution maps that use internal sensors for geo-localization. Other options are available in the Android and iOS application markets. However, these applications always introduce some error margin regarding their measurements, and many of them are not freely available for download and testing [12,13]. The main causes of these errors are two-fold: first, the obvious hardware differences between the smartphones’ microphones and the professional sound level meters; second, the specific algorithms, filters and the sound API that are used to process the sensed values. Thus, a thorough study and comparison of the noise measurement performance when using smartphones becomes necessary.

In this paper, we focus on the main characteristics influencing the design and implementation of reliable systems for the assessment of noise pollution levels using smartphones. We have taken our proposed architecture for crowdsensing-based noise measurements as a starting point, proposing a mobile noise sensing based on off-the-shelf smartphones that offers a performance comparable to the one achieved with professional devices. In particular, the main contributions of our paper are as follows: first, we analyze the behavior of three different algorithms for noise measurement, determining the best approach when taking as reference a professional and fully-calibrated Class II sound level meter [14]. Second, for each algorithm, we evaluate the impact of different sampling rates and sample block sizes. Third, after selecting our candidate algorithm, we make a performance analysis using different types of smartphones, improving the results based on linear regression techniques and then evaluating the optimal time-sampling period. Finally, we validate our proposal in typical outdoor environments. For our tests, we inject pink noise in the range from 35 to 95 dB, matching the dynamic range of the microphones typically available on smartphones. Experimental results show that, with adequate adjustments, it is possible to achieve error margins as low as 0.02%, with a computational overhead of only 15 ms.

This paper is organized as follows: Section 2 reviews the state of the art on this topic. In Section 3, we introduce the different noise measurement strategies studied. Then, in Section 4, we make a detailed analysis of the proposed algorithms. The procedure followed to calibrate our PCE-322A sound level meter in a reverberant acoustic chamber is then detailed in Section 5. Section 6 presents our findings, including an estimation error analysis. A validation of our proposed solution in real environments is then presented in Section 7. Finally, Section 8 presents our conclusions, and future works are discussed.

## 2. Related Works

In the literature, we can find several solutions where smartphones are used as mobile sensing devices. For instance, works such as [4,10,15] propose complete realistic crowdsensing solutions, although providing little detail about the outcome of measurements.

NoizCrowd [16] and Ear-Phone [11] study noise levels using different spatial and temporal interpolation techniques. In particular, Ear-Phone proposes an algorithm that attempts to optimize noise sampling by detecting when the phone is placed in a trouser’s pocket or in a bag.

Later, Pryss et al. [17] proposed a crowdsourcing platform where the microphone of mobile devices is used as an aid for the medical aspects of tinnitus and its treatment. Similarly, Ren et al. [18] use fine-grained techniques to capture the behavior of breathing during sleep through smartphones. In [19], Monge-Alvarez et al. propose an automatic system for the detection of cough based on the standard audio signal of smartphones. They use a local database of sounds of coughing for comparison, and their processing uses emotion recognition algorithms.

Recently, authors such as [12,13] evaluated the quality of noise level measurements of different mobile applications for both the Android and the iOS operating systems. In [12], the authors selected noise levels from 65 to 95 dB in 5-dB increments and generated pink noise within a 20 Hz to 20 kHz frequency range. The results showed that certain sound-measurement applications are inaccurate, thus being unreliable when assessing noise levels via smartphones, especially if using the Android operating system. In [13], five different applications have been evaluated using an Apple iPhone 4s, where the authors evaluated different frequency ranges up to 8 kHz, showing that a large number of applications report wrong levels for loud sounds.

In [20], the authors added external calibrated microphones to the ones provided in smartphones and compared the obtained values against reference measurements. This study evidenced that values retrieved using smartphones’ microphones were considerably lower than those obtained using professional devices.

In [21] uses two components (node-based and crowdsourcing-based) to calibrate smartphones. The node-based component uses a lightweight algorithm to determine the offset using a linear model, doing offline calibration autonomously. Regarding the crowdsourcing-based component, it provides specific calibration parameters for different smartphone models, thereby maintaining a list of the required calibration parameters for each of them.

Our work differs from the previous ones since our goal is to study noise measurement accuracy when only smartphones are used. We developed our own noise measurement application considering current solutions, as well as different algorithms proposed in the literature, and we then selected three different algorithms to determine the noise measuring accuracy with respect to those provided by a professional device. We determine the best configuration option for each of the algorithms in terms of sampling rate and block size. In addition, our study also analyzes which approach provides the best trade-off between accuracy and computational requirements, allowing one to determine the most adequate algorithm for actual application deployment, along with the time sampling period defined for sensing.

## 3. Measuring Noise Level

In this section, we focus on a procedure that leads to accurate noise level measurements using Android-based smartphones. Such a procedure gains special relevance within the framework of our reference crowdsensing architecture for noise data gathering.

### 3.1. Architecture

In a previous paper [22], we proposed a generic crowdsensing architecture. In particular, our proposal integrates two types of elements: Mobile Sensing Noise Clients (MSNC) and Cloud Data Collection Servers (CDCS). Those two elements are connected to each other through a data transmission network.

**Figure 1:**
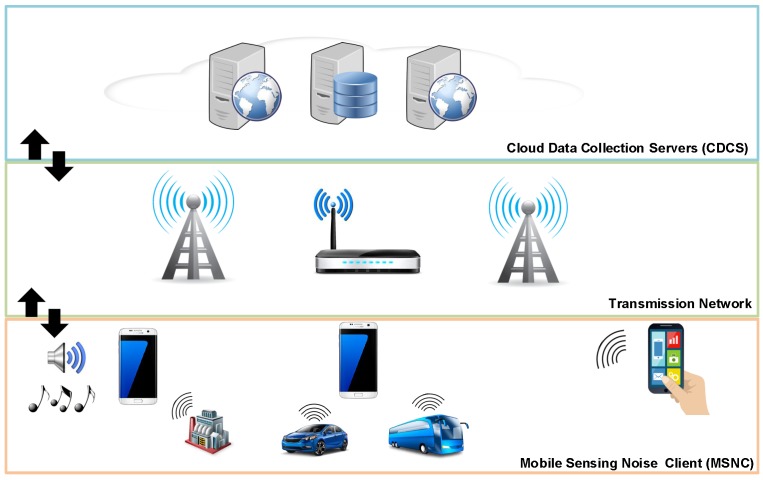
Proposed crowdsensing architecture for noise analysis.

Particularizing our proposed architecture to the scope of the present work, the MSNC is the mobile phone or the set of mobile phones that will provide sound sensing operations by capturing noise data and then delivering that data to the CDCS. Additionally, the CDCS is a single server or a server farm that allows receiving, processing, analyzing and sharing the sensed data. This architecture is shown in Figure 1.

### 3.2. Measuring Background Noise Level

The basic element supporting noise level estimations is the microphone. Nowadays, smartphones incorporate one or more internal microphones, and their level of sensitivity varies according to the smartphones’ brand and model. In addition, the quality of the readings they provide is directly related to characteristics such as the type of filter used (i.e., MaxRF, Enhanced RF), and it is impedance.

In general, once the microphone is activated, it responds to vibrations in the air (sensitivity), converting them into electrical current fluctuations (sound pressure). Afterward, these electrical signals can be evaluated as a signal in the time domain or in the frequency domain. The first approach is a common one, representing the value of the signal as a function of time. The second requires a previous mathematical transformation procedure to switch to the frequency domain, thus introducing additional overhead. In both methods, the sound signal can be processed and its loudness computed over long time intervals.

The International Electrotechnical Commission (IEC) 61672:2002 [23] defined two categories, namely Class 1 and Class 2, for Sound Level Meter (SLM) equipments, defining progressively loose tolerance levels of ±1.1 and ±1.4 dB, respectively. Thus, Class 1 devices are highly accurate, whereas Class 2 encompasses general purpose devices. Usually, based on this standard, the measured noise values are adjusted using filters. All SLM devices apply frequency weightings of Type A, B, C, D or Z [23]. In particular, A-weighting is regularly used because it offers a good correlation with the subjective human perception. In particular, the filter can be applied both in time and frequency domains.

The measured value is expressed in decibels, and the equation is defined as follows:

(1) $$L_{Ap}=10\log_{10}\frac{p_{rms}^{2}\cdot \Delta filter}{p_{ref}^{2}}$$

where $p_{ref}$ is the reference sound pressure and $p_{rms}$ is the root-mean-square sound pressure at a point during a given time interval. In addition, Δfilter refers to frequency-specific A-weighting coefficients [24].

In Equation (2), $L_{A_{eqp}}$ is the average sound level-equivalent for A-weighting applicable to a specific sample block of size BS.

(2) $$LA_{eqp}=10\log_{10}\frac{1}{BS}\sum_{i=0}^{BS-1}\left[10^{\frac{LAp_{i}}{10}}\right]$$

The Sampling Rate (*SR*) or sampling frequency is the number of samples captured per unit of time, being an important factor when calculating environmental noise levels. The higher the sampling rate, the more accurate the voltage fluctuations, and therefore, its shape more accurately resembles the original sound wave. Concerning the Block Size (*BS*), it is the total number of samples stored for processing in a single capture.

### 3.3. Android-Specific Issues

In the Android platform, there are two classes for managing sound resources, i.e., the AudioRecord and the MediaRecorder class. AudioRecord allows data analyzing while recordings are still in progress. Such analysis is dependent on the minimal internal buffer provided by the read object. These raw data are then converted to standard noise levels. On the other hand, when the MediaRecorder is used, data are instead copied to a file, and it provides a method that returns the maximum absolute amplitude that was sampled since the last call. Both classes allow you to configure audio characteristics including sample rate, block size, input and output channels, etc. In our tests, we selected the AudioRecord class.

## 4. Noise Calculation Algorithms

As mentioned earlier, we can find a few free smartphone applications able to measure noise levels, although the majority suffers from large measurement errors. Some of these applications have used a method to calibrate the sampled values. A part of these algorithms also defined a fixed block size and sampling rate.

In this section, we study three different algorithms that allow measuring noise levels, with an emphasis on smartphones. In particular, we have considered three algorithms, where the first one operates in the frequency domain, and the second and third algorithms operate in the time domain. All of these algorithms allow adjusting both the sampling rate and the block size, and they were implemented in our Android-based application described later on.

### 4.1. Fourier-Based Algorithm

This first solution, which is described in Algorithm 1, follows a procedure that consists of retrieving the raw noise measurements and transforming these values from the time to the frequency domain. Specifically, we used the Fourier transform with data previously standardized and processed using a Hamming window. Once the Fourier transform is applied, the data are evaluated and processed using Equation (2), applying the corresponding A-weighting coefficients for the different frequencies. Finally, after a cycle, the noise value is calculated using Equation (2).

### 4.2. Time Domain-Based Algorithm

This second solution, which is described in Algorithm 2, is one of the most widely known and adopted. It processes the noise levels in the time domain as follows: values are read from a set of raw data and are then processed using Equation (1). In this equation, the value of the reference pressure ($p_{ref}$) is set to one. Once the cycle is completed, it calculates the average noise value.

### 4.3. Normalized Time Domain Algorithm

This third algorithm shares the same basic characteristics as Algorithm 2. The main difference lies in the fact that samples are normalized before the noise estimation is made, so that the referential pressure of Equation (1) now becomes $p_{ref}=20$ μPa (0.00002 N/m${}^{2}$), which is the standard reference value for sound pressure.

**Algorithm 1:** dB(A) calculating using Fourier transform.
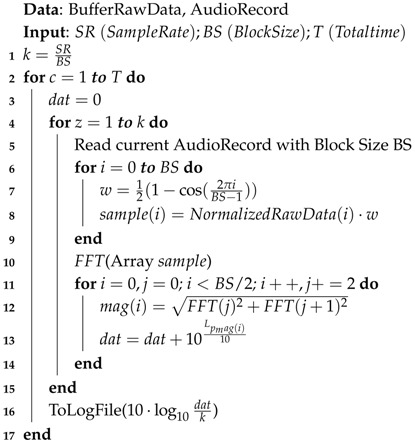


**Algorithm 2:** dB(A) calculation using time series and non-normalized data.
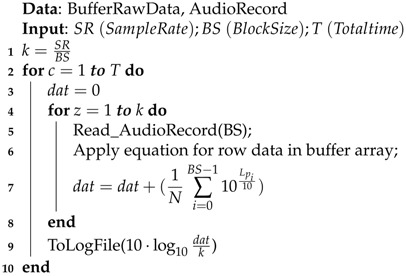


## 5. Calibration Procedure

Our experimental scenario analyzes the accuracy of the three aforementioned algorithms in smartphones, comparing them with the measurements obtained by a professional sound level meter. Specifically, we used the PCE-322A sound level meter [14] for smartphone calibration. This device has a condenser microphone and presents a response to sound pressure in the range between 20 Hz and 20 kHz. It achieves a precision level of ±1.4 dB and complies with the IEC61672-1 standard for Class 2 devices. Concerning the smartphone used for the testing, we selected a Samsung Galaxy S7 Edge (Model SM-G935F) running the Android 6.0.1 operating system. This smartphone incorporates two microphones on the top and the bottom, respectively. The main microphone is of good quality, integrating a low-noise input buffer and active noise cancellation [25]. It is worth pointing out that the microphones included in both professional sound level meters and smartphones are omnidirectional.

**Figure 2:**
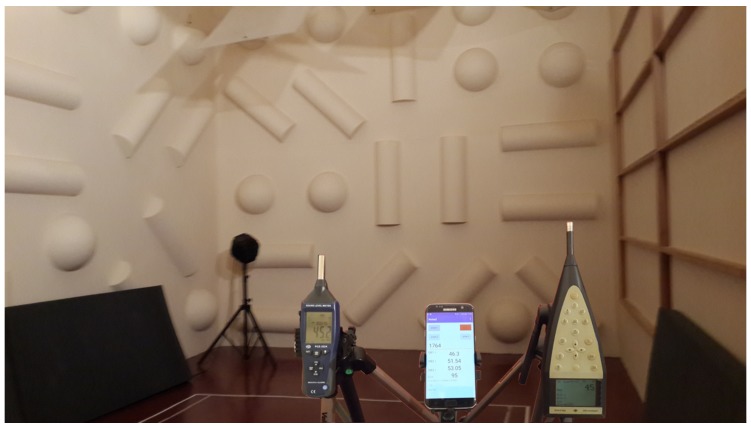
Noise calibration using professional devices. Location: reverberant acoustic chamber, Technical University of Valencia.

To guarantee the highest measurement accuracy, our PCE-322A sound level meter was calibrated using a Class 1 sound level meter in a reverberant acoustic chamber, both of which are institutional resources with limited access and availability. Our purpose was to make sure that both sound level meters provide similar readings, as shown in Figure 2.

In our experiments, the sound source was aligned opposite to the smartphone and the sound level meters. The noise was generated by a Creative Lab T30 2.0 speaker. In particular, we injected pink noise generated by the Audacity software package in the range from 35 to 85 dB (A) in 10-dB steps, which is the typical dynamic range of microphones embedded in smartphones. The sound levels injected were calibrated using the PCE-322A device. Then, in our study, we implemented and tested the three algorithms defined in the previous section using different sampling rates (8 k, 11 k, 16 k, 22 k and 44 k), as well as different block sizes.

For each noise level being evaluated, we took a total of 30 short samples lasting 1 s each, and prior to each sampling procedure, there was an initial warm up period of 15 s to achieve a stable measurement level.

Finally, it is worth mentioning that the algorithms were developed in Android Studio 2.1.3 using the AudioRecord class, and the 16-bit audio format was the one used by default.

## 6. Experimental Results

Once the algorithms have been implemented and tested from a functional point of view, our next goal was to determine the impact of the sampling rate and the block size in the noise estimation accuracy. It should be noted that the sampling rate and the block size affect the accuracy of noise calculations due to two factors: frequency resolution and filtering. The frequency resolution improves as the acquisition time increases. If the acquisition time is fixed, as in our case, a higher sampling rate will allow, according to the Nyquist theorem, to capture the behavior at higher frequencies, being that the maximum measurable frequency is half the sampling rate. This means that frequencies above this threshold are filtered out. However, if the sampling rate is increased, but the block size (number of samples acquired) is maintained, it will correspond to an overall lower acquisition time, and so, the representativeness of measurements will be reduced, again resulting in a lower spectral resolution.

It is also worth pointing out that, in our case, we are not targeting at a very high spectral resolution, nor at encompassing very high frequencies; instead, our goal is to achieve representative noise measurements that mimic the sound level meter results with high accuracy, meaning that lower frequencies are the most representative in terms of noise assessment from the human ear perception system, and so, this is our ultimate goal.

With this purpose in mind, we analyzed the results obtained by the different algorithms against the values stored by the PCE-322A device. In parallel, we analyzed the computation time associated with each algorithm to allow determining the best trade-off between estimated error and processing overhead. In addition, once the candidate algorithm was obtained, we studied the variability when using different types of smartphones and optimized results using linear regression techniques. Finally, we studied the sampling period that offers the best trade-off between sample length and accuracy.

### 6.1. Sampling Rate Analysis

In this section, we analyze the impact of the Sampling Rate (*SR*) on each of the tested algorithms. In particular, we want to determine whether the actual sampling rate significantly affects the noise estimation. For this purpose, we carried out different tests where noise levels were collected from readings using slightly different block sizes (*BS*), where the block size refers to the number of samples gathered so that, combined, the ratio between *SR* and *BS* returns an integer value. As a result, *BS* values are in the range between 1500 and 2500 samples. Then, for each sampling rate and each block size combination, we determined the average noise value for all of the sampling periods, in dBA.

**Figure 3:**
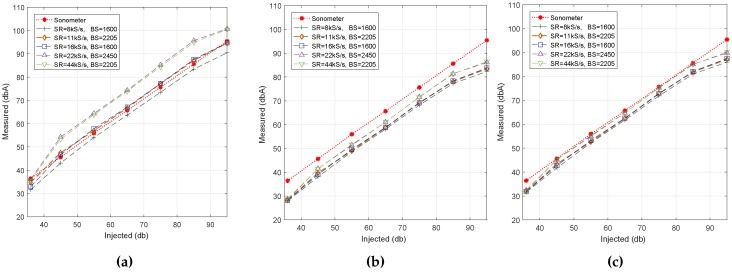
Sampling rate analysis. (**a**) Algorithm #1.; (**b**) Algorithm #2.; (**c**) Algorithm #3.

Figure 3 shows the impact of the selected sampling rates on each algorithm. In general, the three tested algorithms show a linear increase as the input noise level varies. This is desirable, as a non-linear curve within the microphone dynamic range will mean that its quality is poor, possibly being unsuitable for this task.

Concerning the impact of the sampling rate (*SR*) on noise estimations, we find that, in general, values tend to increase when increasing the *SR*. Moreover, we find that Algorithms #2, and #3 show values close to the reference ones when *SR* = 22 kS/s and *SR* = 44 kS/s, respectively, while Algorithm #1 shows better results for *SR* = 11 kS/s. In particular, it can be clearly observed that Algorithm #1 exhibits a significant difference with respect to the reference value when *SR* > 16 kS/s. Overall, we find that Algorithm #3 is the one showing the best match towards the reference values.

### 6.2. Block Size Analysis

In this section, we determine the impact of the block size on noise estimations. To this end, we fixed the sampling rate at 22 kS/s and made several tests with characteristics similar to the previous ones. Specifically, we used six different block sizes to evaluate each algorithm.

**Figure 4:**
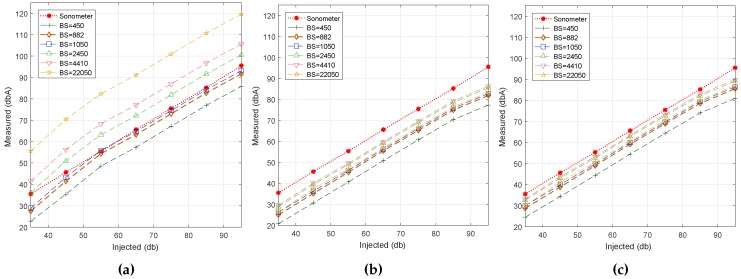
Block size analysis. (**a**) Algorithm #1.; (**b**) Algorithm #2.; (**c**) Algorithm #3.

Figure 4 shows the behavior achieved for the noise range under test using different block sizes. In general, the three algorithms mostly present a linear behavior when the injected noise values increase. We find that Algorithm #3 best fits the reference value when *BS* = 882 samples. Regarding Algorithm #1, we find that the best result is achieved for *BS* = 450 samples. In addition, Algorithm #1 is the only algorithm that experiences a direct relationship between the block size and the estimated noise values (dBA). For Algorithms #2 and #3, this relationship was not so clear, although the lowest *BS* size still provides the lowest noise estimations.

### 6.3. Algorithms Comparison when Fixing the Block Size

In this section, we want to extend our evaluation to the analysis of the impact of using different sampling rates when a same block size is adopted for all cases. In particular, we have assigned a default block size of 2012 samples to all measurement setups, which approximately corresponds to the mean size of the blocks evaluated in the previous section. In addition, we have discarded Algorithm #2 since the values it returned in the previous section were the most distant ones to our reference.

**Figure 5:**
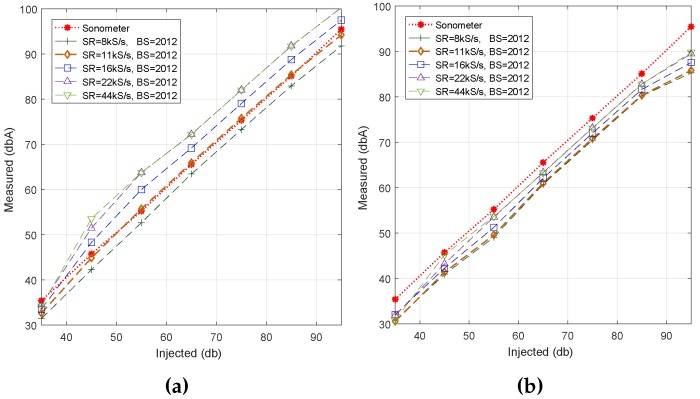
Sampling rate analysis when fixing the block size. (**a**) Algorithm #1; (**b**) Algorithm #3.

We consider that it is interesting to compare the behavior of Algorithms #1 and #3 due to their different noise calculation approaches, that is Fourier transform vs. time domain analysis, respectively. The samples were obtained with the same smartphone used for the previous tests, and the average value corresponding to 30-second periods was calculated for each noise level (35 to 95 dB) injected. Figure 5 shows the impact of the sampling rate on the noise estimation accuracy. We find that, in general, there is an increasing behavior with *SR*, meaning that increasing *SR* values causes the measured noise values to increase, as well. We also noticed that, for Algorithm #1, a nearly optimal curve adjustment is achieved when using a sampling rate of *SR* = 11 kS/s, whereas for Algorithm #3, all of the returned values are slightly below the reference line provided by the professional sound meter.

### 6.4. Estimation Error ($\epsilon_{s}$) vs. Computational Overhead

We now proceed to analyze which algorithm exhibits the lowest error with regard to the reference value, as well as their impact on the computational overhead. To calculate the error, we used a total period *T* = 30 s, with $\frac{SR}{BS}$ sample blocks per second, adopting the same characteristics as in the experiments detailed above.

Concerning the error value, it was calculated using Equation (3):

(3) $$\epsilon_{s}=|\frac{i_{s}-R_{s}}{R_{s}}|$$

In this equation, $\epsilon_{s}$ represents the relative average error for sample *s* with regard to the set of reference data; $i_{s}$ represents the value calculated by the algorithm for sample *s*; and $R_{s}$ represents the reference value of the sound level meter for sample *s*.

With regard to the calculation of the computation time, we obtained the average value corresponding to 1000 independent executions for each particular algorithm under the sampling rate and block size defined.

Table 1 presents a summary of the mean and maximum estimation errors achieved, along with the corresponding computational overhead. This table is completed based on the results obtained in the previous evaluations for different sampling rates and block sizes, but we have filtered this table showing the three best results obtained in each algorithm. Notice that Algorithms #1 and #3 have a mean error value of about 1 to 4% and that they introduce a similar overhead. On the other hand, the computation overhead for Algorithm #1 is higher due to the need to perform the Fourier transform prior to determining the error. Additionally, we find that the maximum error for Algorithms #3 and #2 is higher (10%) when compared to the first algorithm.

To have a greater insight into the different trade-offs faced, Table 1 shows the *SR/BS* combinations returning the lowest average error with respect to the sample rates evaluated, along with the respective computation overhead. There is evidence that Algorithm #3 offers the smallest error margins, achieved for *SR* = 44 kS/s and *SR* = 22 kS/s when having *BS* = 2205 and *BS* = 2450, respectively, achieving an error of less than approximately 4%. Algorithm #2 presented errors in excess of 4%. Finally, regarding Algorithm #1, it is indeed the one offering the best combination by achieving both low error values and low computational overhead, and so, it is considered as the best candidate solution.

### 6.5. Analysis Using Different Smartphone Models

**Figure 6:**
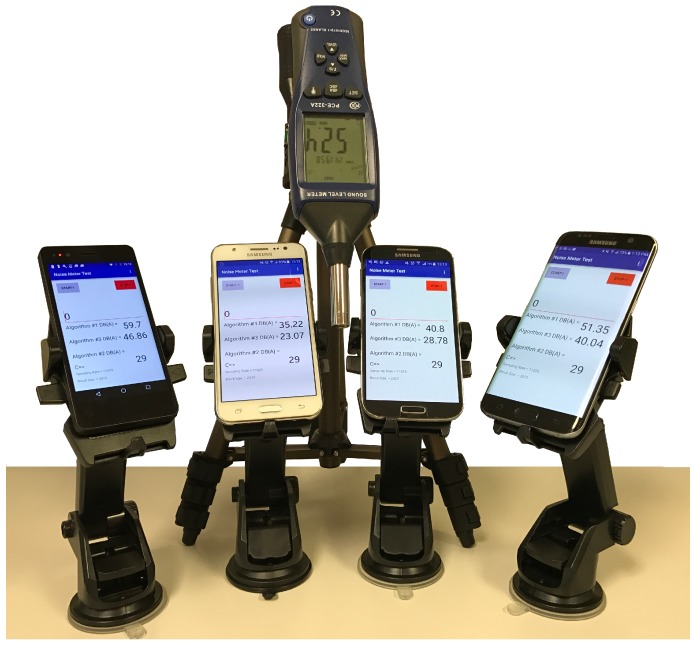
Smartphone models used for testing. From left to right: BQ Aquaris, Samsung J5, Samsung S4 and Samsung S7 Edge.

Once Algorithm 1 was found to be the best option, the next task was to evaluate the effectiveness of the selected algorithm when obtaining samples using different smartphone models. Figure 6a shows the smartphone models evaluated. All smartphones run the Android 6.0 operating system and are able to correctly run the developed application.

In the experiment, we performed a procedure similar to that followed in previous tests. In particular, we injected pink noise in the range from 35 to 95 dB, and each test lasted 30 s.

**Figure 7:**
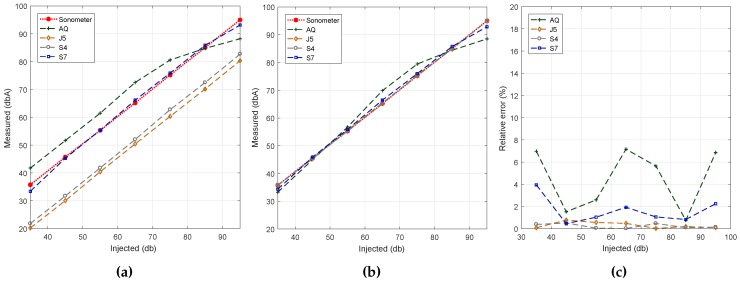
Estimation accuracy for the different smartphone models with and without linear regression. (**a**) Algorithm #1: default sampling; (**b**) Algorithm #1: values adjusted using linear regression; (**c**) Algorithm #1: estimation error.

Figure 7a shows the obtained results. In general, we find that, except for smartphone model BQ Aquaris (AQ), all other smartphones models (S4, J5 and S7) present a linear behavior. In terms of result accuracy, though, we find that only the results for the Samsung S7 device are near the reference values. Such near-optimal accuracy is expected since the experiments performed earlier on relied on this same device. Thus, we find that the results achieved using the proposed algorithm show model-specific variations, which are in general expected due to hardware differences.

To solve the problem detected, our next step was to apply linear regression techniques with respect to the reference dataset. Our goal was to adjust the results achieved with the different smartphone models so that they resemble the reference ones as much as possible. The results of this curve adjustment process are shown in Figure 7b. It quickly becomes evident that the output results for most of the models fully agree with the reference value, with more pronounced differences for the BQ Aquaris smartphone case in the range from 65 to 75 dB (A). Finally, Figure 7c shows that, after the adjustment procedure, the error is less than 2% in Samsung phones, the BQ Aquaris model being the one showing the highest error values. Anyway, the error is always less than 8%, which is a reasonable value.

### 6.6. Analysis for the Same Smartphone Model

In this section, we compared the differences between smartphones of the same model/provider and determine the differences between them. However, differences between smartphones of the same model are expected, especially for low-range market devices, where cheaper hardware is used. In particular, we picked four BQ Aquaris smartphones for our tests since these are the cheapest ones used.

Figure 6a shows the results for our noise sensing tests before applying any curve adjustment. The experiment was performed under the same conditions used before. We can see that there are some differences between smartphones, although the shape of the curve is similar in all cases, with a loss of linearity for values above 75 dB.

**Figure 8:**
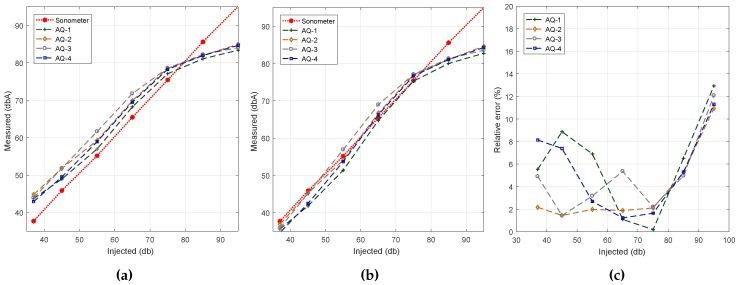
Estimation error analysis when using similar smartphones. (**a**) Before regression; (**b**) after regression; (**c**) estimation error.

Figure 8b shows the output after performing the linear regression procedure. We can now see how values tend to better resemble the reference values, showing differences for inputs of 85 dB and above due to the non-linearity detected earlier. Finally, Figure 8c shows the value of the estimation errors, which are below 8% in most of the cases.

### 6.7. Impact of Reducing the Sampling Period

**Figure 9:**
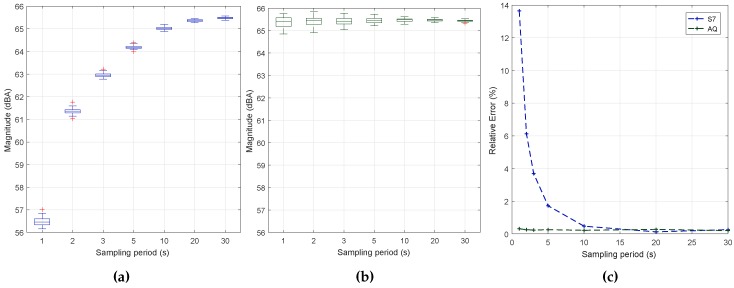
Sampling period analysis. (**a**) S7 edge; (**b**) BQ Aquaris; (**c**) estimation error.

To complete our study, our next goal was to determine the optimal duration of the data collection process. This is a critical issue when aiming at minimizing the resource usage of smartphones. With this purpose, we have evaluated the impact of the sampling time for two smartphone models: the low-end BQ Aquaris and the high-end Samsung S7 Edge. For both devices, we have obtained 30 samples for each sampling period tested. In all cases, the block size used is 2012, and the sampling rate is 11 k, as defined in previous sections. The injected noise was fixed at the intermediate value of 65 dB in all of the readings. Figure 9 shows the sample estimations achieved with both smartphones when varying the sampling period, along with the associated error values. In particular, Figure 9 shows that the Samsung S7 edge smartphone introduces a measurement bias detectable when the interval is very low, meaning that a filter is introduced that causes values to slowly converge to the final value. This effect is not noticeable in the BQ Aquaris smartphone, as shown in Figure 9, which is much more linear. In both cases, we find that, as expected, higher sampling periods are associated with a lower result variability, although the margin of variation typically remains below ±0.5 dB in all cases. Figure 9 shows the error towards the reference values for the two smartphones under tests. Clearly, the S7 smartphone introduces an error that only becomes negligible when the overall sampling period is greater than 10 s. Therefore, it is important to determine the required stabilization time for the different smartphones models.

Based on the prior analysis, we decided to broaden our study to gain further insight into the sound capture process for the initial samples taken during the first second and thereby determine the duration of the transient period detected above. This procedure was repeated 30 times.

**Figure 10:**
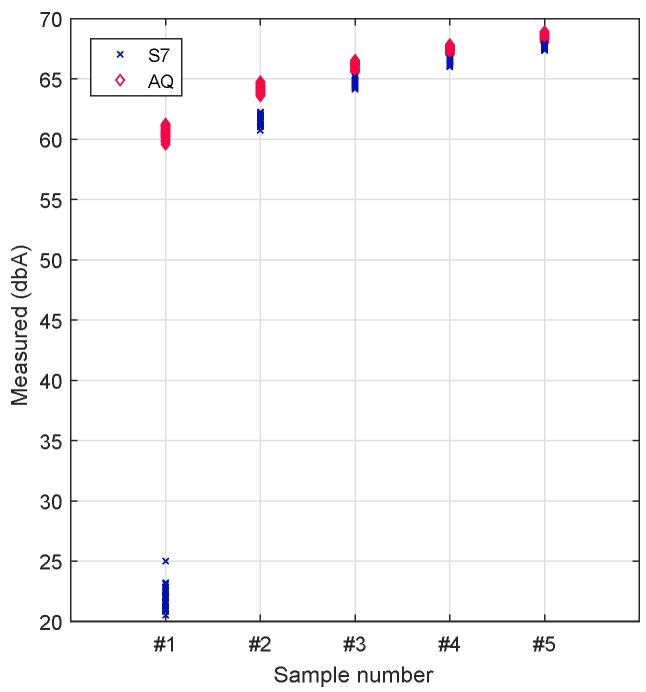
Analysis sampling S7 and Aquaris for 1 s.

Figure 10 shows that, during this initial second of sampling, sample values gradually tend to increase, initially taking values below the reference value (65 dB) and then becoming slightly higher. The main problem detected occurs for the first sample taken with the Samsung S7 smartphone, where the values registered are more than 40 dBs below the reference value, thus causing a substantial bias when averaging these different samples. Given these circumstances, at least the first sample taken should be discarded in order to increase the reliability of the results and to achieve meaningful measurements in a short time period.

**Figure 11:**
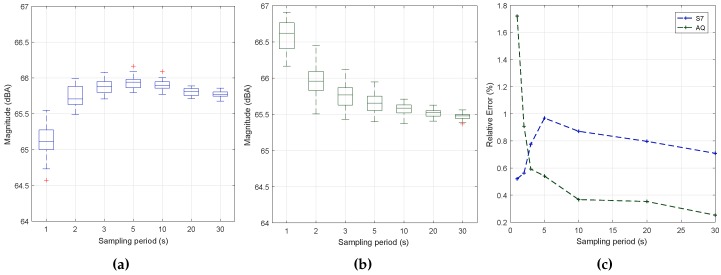
Sampling period and error analysis when removing the first sample. (**a**) S7 Edge; (**b**) Aquaris; (**c**) error analysis.

Figure 11a,b shows the new results when varying the sampling rate and discarding the first sample during this initial second. We find that there is a substantial prediction improvement, especially for the Samsung S7 device, which was the one experiencing the problem. We also find that the readings do not depend significantly on the period of time chosen.

Figure 11c now shows that the margin of error is below one percent for most of the cases. The worst result takes place when using a sampling time of one second using the BQ Aquaris model; anyway, the average error remains below 2%, which is considered acceptable for these types of measurements.

Overall, based on the results obtained, we can conclude that Algorithm #1 is an optimal candidate when used together with the following conditions: discard the first sample; adopt a sampling rate of 11 k; select a block size of 2012 (evaluated); and apply a curve adjustment (linear regression) specific to each smartphone model. This strategy allows reducing the sampling error below 1% in most cases, even when limiting the sampling period to a very short interval of 1 to 2 s. Finally, by analyzing Table 2, we show that the mean value obtained is close to the reference value generated for testing (65.5 dBA). We also find that, on both smartphones, the standard deviation experienced a moderate decrease when increasing the sampling time.

## 7. Validation in Real Outdoor Environments

All of the aforementioned results were obtained in a controlled noise environment. However, field noise measurement results may experience significant differences due to environmental effects, such as humidity, temperature and the stability of the device, among others. Therefore, to complete our study, our next goal was to validate the behavior of the candidate algorithm in real environments, comparing the results obtained with those of our professional noise level meter.

**Figure 12:**
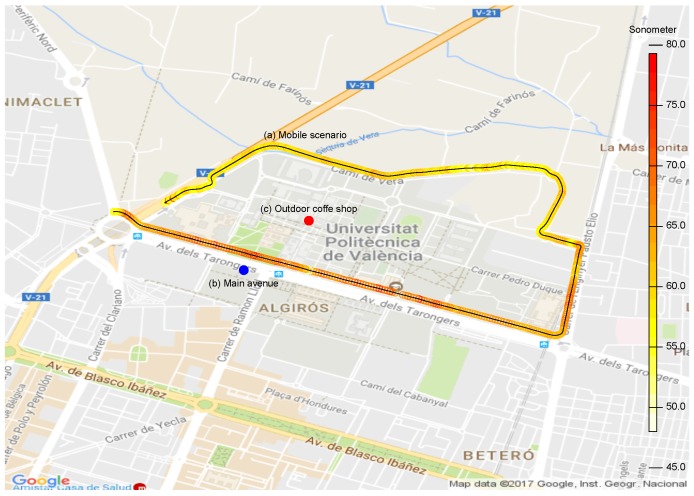
Analysis for the mobile scenario.

We decided to perform our study in three different outdoor conditions: the first experimental results where obtained under mobility with the support of a vehicle; the second set of results was obtained when statically positioned near a main avenue; and the third set of results was obtained at a crowded outdoor coffee shop. Figure 12 shows the locations associated with the three scenarios we used for validation.

**Figure 13:**
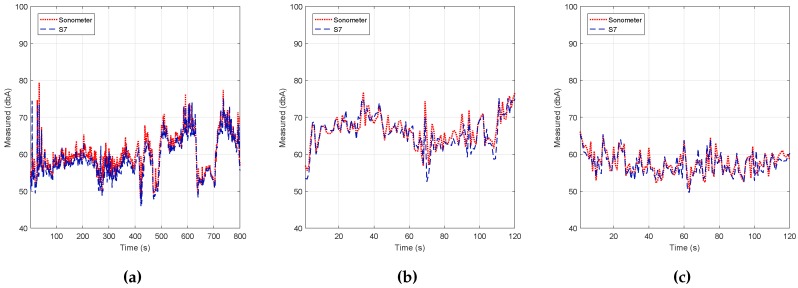
Analysis noise pollutions for three scenario outdoor environment. (**a**) Mobile scenario; (**b**) main avenue; (**c**) outdoor coffee shop.

In detail, the first set of results was obtained by having the noise level meter and the Smartphone S7 in a car with the windows down, both devices being statically positioned with an appropriate holding arm. We then followed the path shown in Figure 12 at an average speed of 20 km/h, the obtained results being shown in color in Figure 12 and graphically in Figure 13a. The latter results showed a behavior close to the ones obtained with the sound level meter with a margin of error of 0.0308% (see Table 3).

Regarding the second set of tests, we installed the equipment (noise level meter and smartphones) on one side of a main avenue (Avenida Tarongers) in such a way that it remained static for the whole test duration. The measurements were taken at 17:00, a time when the traffic density was moderate. The results are shown in Figure 13b and Table 3, and again, we can find a high resemblance when compared to the results produced by the professional sound level meter.

With respect to the third set of tests, the test equipment was installed on the table of an outdoor coffee shop within our university campus following a similar procedure. The results are shown in Figure 13c and Table 3. In particular, we find that the error margin was even lower compared to the previous results, with an error value of only 0.0257%.

Overall, we consider that the results obtained are quite satisfactory and evidence the adequateness of the proposed solution, as well as of the methodology adopted.

## 8. Conclusions and Future Work

The widespread adoption of smartphones, and the various sensors they integrate, offers unlimited potential waiting to be unleashed. In particular, the combination of multiple smartphones acting as mobile sensing devices allows achieving massive monitoring, a process known as mobile crowdsensing.

In this paper, we proposed using the microphones of smartphones as sensors to create noise level maps for metropolitan areas. In particular, we address the issue of measurement accuracy and representativeness when using the microphones of commercial smartphones. To this aim, we implemented and compared different algorithms typically used to obtain Type A noise levels, assessing the impact of the sampling rate and the buffer size on noise measurement accuracy. We also evaluated the impact that different issues could have over the measurement process: (i) the result comparison when testing with different smartphone models; (ii) the result comparison when testing different smartphones of the same model; (iii) the model-specific tuning by applying linear regression techniques; and (iv) the analysis of the trade-off between sampling period duration and noise estimation error.

Experimental results show that both the sampling rate and the selected buffer size can have a significant impact on the accuracy of noise level estimations, being that estimation errors can vary from 1% to 12% in the best cases. However, although statistically representative, we consider that errors between 1 and 2 dB are acceptable for noise measurement in the majority of the scenarios. More important, we find that, if an adequate selection is made, it is possible to combine low noise-level errors with a low computational overhead, a situation that is ideal in crowdsensing contexts. In addition, we show that low-end smartphones are prone to introduce a higher error than high-end smartphones on average, although the additional filters included in the latter may require some of the initial measurements to be discarded. Overall, we were able to demonstrate that it is possible to accurately assess noise levels by taking relatively short samples (from 1 to 3 s) while introducing a minimal estimation error.

As suggestions for future algorithm developments, we consider that it is important to take into account the differences between mobile phones from different manufacturers, which are mainly noticeable in terms of microphone quality and the presence of filters; the latter can cause unwanted effects, such as initial transient periods in the measurements obtained. Similarly, the choice of a specific configuration in terms of sampling rate and block size will have an effect on the noise measurements made and should also be taken into account. Finally, the algorithms developed should try to compensate for non-linearities in noise measurement over the different frequency ranges.

As future work, we plan to integrate this algorithm in a crowdsensing application to achieve distributed noise measurements. Additionally, we plan to evaluate other smartphones sensors (i.e., gyroscope, accelerometer, orientation, GPS) to determine the ideal time to capture environmental noise in urban areas.

## Figures and Tables

**Table 1:** Estimation error and computational overhead achieved with the different algorithms (best overall configurations).

Sampling Rate (kS/s)	Block Size	Algorithm	$\bar{\epsilon_{s}}$ (%)	$\max(\epsilon_{s})$ (%)	Overhead (ms)
11	2012	1	0.01931	0.07796	25.1533
11	2205	1	0.02420	0.04589	25.3667
16	1600	1	0.03236	0.09625	25.7133
44	2205	3	0.03146	0.11664	25.4101
22	2450	3	0.03154	0.11048	16.0122
44	2012	3	0.04579	0.12980	25.0178
44	2205	2	0.09326	0.21741	25.3252
22	2450	2	0.09334	0.21124	16.1289
11	2205	2	0.12582	0.22328	11.1083

**Table 2:** Sampling period analysis (when removing the first sample).

Smart Phone Type	Sampling Period (s)	Mean (dBA)	$\bar{\epsilon_{s}}$ (%)	Std. Dev.
Samsung S7 Edge	1	65.1229	0.0052	0.2355
	2	65.7344	0.0056	0.1382
	3	65.8856	0.0078	0.0979
	5	65.9405	0.0097	0.0890
BQ Aquaris	1	66.5757	0.0172	0.2270
	2	65.9599	0.0091	0.2128
	3	65.7659	0.0059	0.1880
	5	65.6615	0.0054	0.1506

**Table 3:** Error in the outdoor real environment.

Scenario	$\bar{\epsilon_{s}}$ (%)	$\max\left(\epsilon_{s}\right)\;\left(\%\right)$
(a) Mobile scenario	0.0308	0.2275
(b) Main avenue	0.0260	0.1989
(c) Outdoor coffee shop	0.0258	0.1151
